# A Unique Case of Asystole Secondary to Facial Injury

**DOI:** 10.1155/2012/382605

**Published:** 2012-02-12

**Authors:** Siddharth A. Wartak, Reshma A. Mehendale, Amir Lotfi

**Affiliations:** ^1^Division of General Internal Medicine, Baystate Medical Center, Springfield, MA 01199, USA; ^2^Ophthalmology Department, New York Eye and Ear Infirmary, NY 10003, USA; ^3^Division of Cardiology, Baystate Medical Center, Springfield, MA 01199, USA

## Abstract

Bradycardia has been reported during intraoperative settings of craniofacial, cerebropontine angle and trigeminal ganglion surgeries (Schaller et al. (2009, 1999); Parbhakar et al. (2009); Koerbel et al. (2005); Roberts et al. (1999)). It is also commonly seen in children undergoing orbital and maxillary fractures repair. This mechanism has been described as the trigeminocardiac reflex (TCR) (Schaller et al. (2009, 2004); Kim et al. (2000); Lang et al. (1991); Van Brocklin et al. (1982)). We report an unusual case of posttraumatic bradycardia and recurrent asystole in a previously healthy adult patient from possible TCR in the absence of any surgical intervention to the head and orbital area.

## 1. Case Presentation

A 56-year-old healthy male presented to the emergency room with facial injuries following an accident while riding his motor scooter. He was wearing a helmet and did not suffer from a head injury or loss of consciousness. He was alert and oriented on presentation; on examination he had ecchymosis around his eyelids and a lacerated left eyebrow. His systemic and neurological examination was normal. His complete blood count and complete metabolic panel were within normal limits and toxicology screen was negative. He had a sinus bradycardia with a heart rate of 50–60 beats per minute with normal blood pressure. The electrocardiogram showed sinus bradycardia with normal PR interval. CT head, cervical, chest, and abdomen studies were normal. Maxillofacial CT showed undisplaced fracture of the left zygomatic arch with intact orbits. He had a left temporal artery laceration which was sutured and pain was controlled with opioids. 

**Figure 1:**
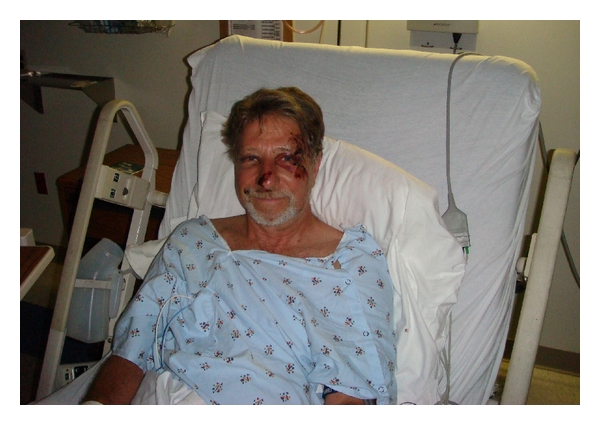
Day 1.

**Figure 2:**
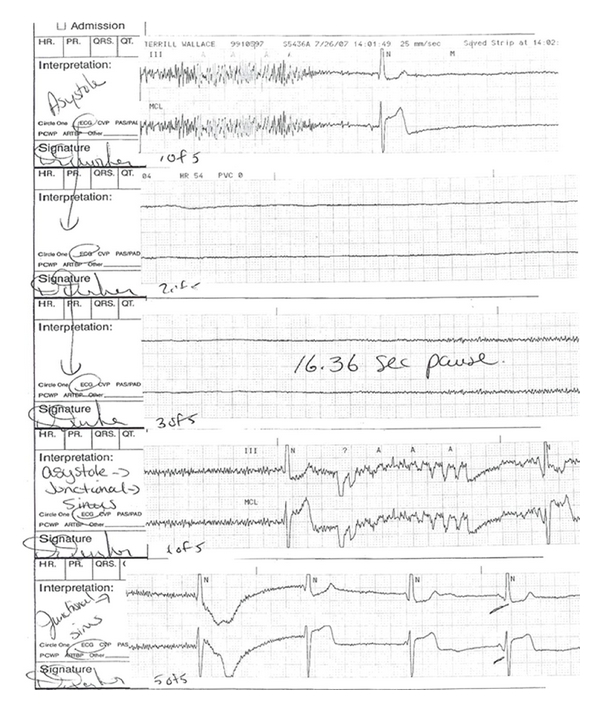
Cardiac telemetry reading showing asystole for 16.36 seconds.

Due to the patient's sustained bradycardia, he was admitted to a telemetry bed for continued observation. The following morning, he had a 16-second pause and became unresponsive but regained consciousness before resuscitation could be initiated (Figures 1 and 2). In view of this event, the patient was transferred to the cardiac intensive care unit and observed with transcutaneous pacer pads in place. Over the next 24 hours, he had 3 more episodes of asystole (Stokes-Adams) lasting 3–6 seconds. A transthoracic echocardiogram preformed to evaluate for evidence cardiac contusion was found to be normal. Cardiac biomarkers were obtained and were nonelevated.

**Figure 3:**
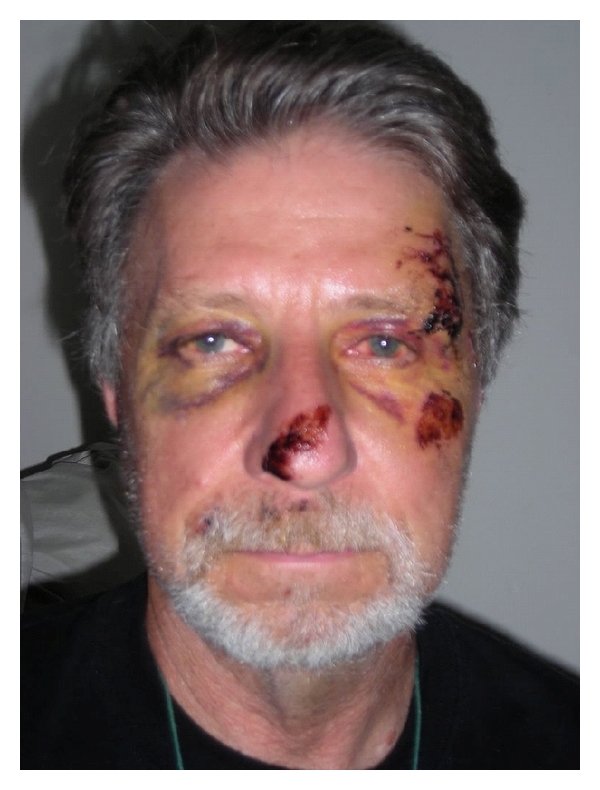
Day 4.

The cause of his significant bradycardia and recurrent asystole was a diagnostic quandary. Our patient was not on any bradyarrhythmic medications, his urine toxicology was negative, and there was no history of any tick bite. Over the next 48 hours, his frequency and duration of asystole decreased and his bradycardia gradually resolved. His opioid medications for pain control were gradually decreased over this period. He did not require any interventions like transdermal or transvenous pacing. After removal of his sutures for temporal artery laceration and no new onset of symptoms, the patient was discharged on day 4 of hospitalization (Figure 3). Over the past 2 years of followup since this episode, the patient has been on no medications and has had no recurrence of his symptoms.

## 2. Discussion

We report a rare case of persistent bradycardia and recurrent asystole (Stokes-Adams) in a previously healthy individual following a traumatic facial injury. Our patient was asymptomatic prior to the incident and not on any medications. Complete history, examination, and lab workup did not show any cause to explain his persistent bradycardia. Infiltrative diseases such as amyloidosis, scleroderma, and hemochromatosis were unlikely cause in him considering his previous health with no clinical signs. Pericardial disease and acute epicardial coronary disease were excluded with normal echocardiogram, symptoms, and ECG and negative cardiac biomarkers. Patient had no history of any tick bite, and the fact that his symptoms resolved over 3-day period made the Lyme carditis highly unlikely. The new onset of bradycardia which presented following his facial injury and improved during the course of his recovery was an intriguing finding in this patient. 

A possible physiologic explanation is the trigeminocardiac reflex (TCR) [1–6] which can cause a sudden onset of bradycardia and hypotension due to central or peripheral stimulation of the sensory branches of the trigeminal nerve. The afferent tract is one of the three divisions of trigeminal nerve which synapse with the visceral motor nucleus of the vagus nerve located in the brain stem [4]. The efferent tract is the vagus nerve from the medulla to the heart, causing sinus bradycardia with or without junctional escape; junctional rhythm, AV block, bigeminy and nodal beats. This bradycardia can also progress to sinus arrest, asystole, or ventricular fibrillation [7, 8]. 

The oculocardiac reflex (OCR), a subtype of TCR was first described in cats and rabbits in 1870 [9] and is a recognized occurrence during ocular surgery and manipulations of the orbit and its surrounding structures [10–15]. TCR is a relatively less-known brainstem reflex and has been described by Schaller [1, 2, 16]. Clinically it has been reported to occur during intraoperative settings of craniofacial, cerebropontine angle [1, 16, 17], trigeminal ganglion surgery [18, 19], orbital repairs, and during manipulation of maxillary fractures [4]. Opiates, high vagal tone, and pain can further predispose for bradyarrhythmias [20].

Our patient was an athlete and possibly had a high vagal tone. He had an undisplaced fracture of the maxillary bone along with temporal artery laceration. This along with soft tissue swelling could have contributed to irritation and stimulus for the trigeminal nerve causing trigeminocardiac reflex and asystole. This theory can be supported by the observation that over the next few days, as the swelling in the trigeminal area reduced, the patient's bradyarrhythmia improved as well. We believe TCR is a benign process and can be more pronounced in susceptible individuals with high vagal tone. Conservative management is sufficient for treating this rare, self-limiting complication in posttrauma patients, but caution and observation are warranted in the initial phase as asystole may require intervention.
